# A novel meta‐analytical approach to improve systematic review of rates and patterns of microevolution

**DOI:** 10.1002/ece3.3116

**Published:** 2017-06-20

**Authors:** Lucas D. Gorné, Sandra Díaz

**Affiliations:** ^1^ IMBiV, UNC, CONICET, FCEFyN Córdoba Argentina

**Keywords:** contemporary evolution, haldanes, LRI‐framework, phenotypic evolution, plant microevolution, quantitative traits

## Abstract

A classic topic in ecology and evolution, phenotypic microevolution of quantitative traits has received renewed attention in the face of rapid global environmental change. However, for plants, synthesis has been hampered by the limited use of standard metrics, which makes it difficult to systematize empirical information. Here we demonstrate the advantages of incorporating meta‐analysis tools to the review of microevolutionary rates. We perform a systematic survey of the plant literature on microevolution of quantitative traits over known periods of time, based on the scopus database. We quantify the amount of change by standard mean difference and develop a set of effect sizes to analyze such data. We show that applying meta‐analysis tools to a systematic literature review allows the extraction of a much larger volume of information than directly calculating microevolutionary rates. We also propose derived meta‐analysis effect sizes (*h*,* LG* and *LR)* which are appropriate for the study of evolutionary patterns, the first being similar to *haldanes,* the second and third allowing the application of a preexisting analytical framework for the inference of evolutionary mechanisms. This novel methodological development is applicable to the study of microevolution in any taxa. To pilot test it, we built an open‐access database of 1,711 microevolutionary rates of 152 angiosperm species from 128 studies documenting population changes in quantitative traits following an environmental novelty with a known elapsed time (<260 years). The performance of the metrics proposed (*h, LG* and *LR*) is similar to that of preexisting ones, and at the same time they bring the advantages of lower estimation bias and higher number of usable observations typical of meta‐analysis.

## 1 INTRODUCTION

Rates of evolutionary change are important in understanding patterns of evolution and their underlying processes (Gingerich, 2009; Hunt, 2006, 2012; Kinnison & Hendry, 2001). Contemporary evolution, i.e., evolution in ecological timescales, is now widely accepted (Shefferson & Salguero‐Gómez, 2015). Evolutionary change has been shown to feed back, both directly and indirectly, into demographic, community, and ecosystem processes (Bassar et al., 2010; Carroll, Hendry, Reznick, & Fox, 2007; Hairston, Ellner, Geber, Yoshida, & Fox, 2005; Hendry, 2016). In addition, there is a growing body of evidence showing the importance of contemporary evolution in the conservation and management of species, communities, ecosystems, and their societal benefits in the face of accelerated environmental change (Alexander, Vonlanthen, & Seehausen, 2016; Darimont et al., 2009; Merilä & Hendry, 2014).

Several authors have developed tools to measure the amounts and rates of phenotypic change and interpret them (Gingerich, 1983, 1993; Haldane, 1949; Hendry & Kinnison, 1999; Hunt, 2006, 2012). Haldane (1949) proposed the use of the standard deviation of a trait as unit of change within or between populations. He also proposed the use of generations as a natural unit of time, complementary to the use of absolute timescales (years). Gingerich (1993) later used these suggestions to formalize a metric of evolutionary rate (expressed in *haldanes*), which is the difference between populations as units of pooled standard deviation divided by the number of generations of separation between such populations (see equation (1)). The *haldane*s numerator is the total amount of change accumulated between populations in that period. Another common way of expressing evolutionary rate is *darwins* (*(ln(x2)‐ln(x1))/t = ln(x2/x1)/t*) (Haldane, 1949), which expresses the change, in a elapsed time, relative to the initial value of such trait. The response ratio (*lnRR*), an effect size (ES) from meta‐analysis (Hedges, Gurevitch, & Curtis, 1999), could be used to construct an ES analogous to *darwins* is. However, we do not use *darwins* in the present work because in the construction of the database we used years as unit of time, which makes our rate and *darwins* more similar and arguably redundant; note that according to previous reports *darwins* and *haldanes* are positively correlated (Bone & Farres, 2001; Hendry & Kinnison, 1999). Moreover, because different traits from different species are analyze together, *haldanes* is a more appropriate measure because it expresses all changes in a scale‐independent common unit, the standard deviation of the variables (*SD*).

Gingerich (1983) also developed a graphical and statistical framework of analysis (LRI) which allows inference of evolutionary processes from the temporal pattern of evolutionary rates. Briefly, the LRI‐framework is the linear regression between the log_10_ of the absolute values of all rates of change in a temporal series (or the rates of change of a random population of independent, but identically distributed trajectories) and the log_10_ of the elapsed time between samples used to compute the rates of change. This analysis provides information about the “intrinsic” rate of change (i.e., 10 exp {the intercept of the regression}) and the predominant mode of evolution (directional change, random change, or stasis). The slope of the linear regression is expected to be zero if change is predominantly directional, −0.5 if change is not different from an unbiased random walk, and −1 if stasis predominates (Gingerich, 1993, 2009).

The LRI‐framework has received major criticism, focused on its accuracy in estimating “intrinsic” rate of change (Hunt, 2012) and also on the interpretation of the slope (Sheets & Mitchell, 2001). Hunt (2006, 2012) shows that LRI estimates accurately the “intrinsic” rate of change only in case of directional change. Hunt (2006, 2012) provides some tools that complement the inference about evolutionary mode made by LRI, which are applicable to our present work. According to Hunt (2012), rate of evolution, like *haldanes*, is an estimator of the parameter μ_s_ (mean of the generalized random walk, which models directional change); *haldane*'s numerator is a divergence measure, with similar properties as ω (variance of his model of stasis). In the Hunt's approach, it is expected that in a situation of directional change, log_10_(μ_s_) has zero slope with log_10_(time), whereas log_10_(ω) has positive slope; in a situation of unbiased random walk, it is expected that log_10_(μ_s_) has negative slope with log_10_(time) and log_10_(ω) has positive slope; in a stasis situation, it is expected that log_10_(μ_s_) has negative slope with log_10_(time), whereas log_10_(ω) has zero slope. According to Hunt's method, the “intrinsic” rate of change should be estimated using the model of evolution which best fits the data, whereas the LRI model by definition assumes directional change.

In addition, Sheets and Mitchell (2001) argue that the LRI method has lower sensitivity to detect stasis or directional change from random walk than originally claimed, due to the large range of LRI rates produced by random walks. However, we disagree with this last statement because the expected value for the LRI slope in an unbiased random walk (−0.5) is a theoretical value (Bookstein, 1987) not a random variable, so that the test of hypothesis should be as be proposed by Gingerich, 1993 and not by Sheets and Mitchell.

Despite the fact that the theoretical framework for the study of microevolution in quantitative traits was organized by Hendry and Kinnisson already in 1999, few empirical studies have used it, or taken into account its methodology. As a consequence, most reviews of microevolution of quantitative traits in plants have been constrained by the scarcity of reports of evolutionary rates (or the information needed to compute them, i.e., the raw data, or means, sample sizes and *SD*, or coefficients of variation—CV). Bone and Farres (2001) considered only 78 observations from 26 species (of which three were perennials) and mentioned the difficulty of drawing definitive conclusions on the basis of such a small number of studies. A subsequent review by Westley (2011) included a larger empirical database of taxa, but for plants it only included the data published by Bone and Farres (2001). Crispo et al. (2010) analyzed evolution of phenotypic plasticity in response to anthropogenic disturbance, bringing together 212 outcomes from 13 plant species. These authors take the reported evolutionary rates or compute them from the information mentioned above. However, as we show below, an important proportion of the relevant literature does not allow these procedures. This scarcity of systematized data contrasts with the rapidly growing number of studies comparing changes in plant quantitative traits. Another issue in carrying out evolutionary syntheses, highlighted by Crispo et al., 2010, is the need to weight the outcomes according to the strength of their underlying sources of data and also to control pseudoreplication caused by considering several outcomes from the same work and species. We thus set out to devise a method that could take advantage of all these insights and bodies of data.

The first goal of this article is the development of a set of ES which give similar information as preexisting microevolutionary metrics, but allow the extraction of much more information from a systematic review of the literature. Our novel use of well‐tested meta‐analysis methods (Gurevitch, Curtis, & Jones, 2001) has three major advantages. First, the meta‐analytical ES standardized mean difference (SMD) is mathematically equivalent to the *haldane’*s numerator, and, within certain limits, SMD is a close approximation to Gingerich's (1993) *haldane’*s formulation (with some specificities, see below), so that the information provided is similar to preexisting microevolutionary metrics. At the same time, the interconversion between different ES allows the extraction of information from data sources that are not directly comparable (e.g., descriptive statistics such as mean, *SD*, sample size, *F*‐statistic, Pearson moment), so that more information may be incorporated than it was possible in previous reviews. Second, as in any meta‐analysis, it works with a standardized variable (the ES), and to weighs each study by its precision, in the global analysis (1/variance, here variance is the square of the standard error of the ES, which combines sample size and (un)balance of the sampling, but not the variance of the variable). This makes it possible to compare different studies in a quantitatively manner and use them together in a single analysis, controlling for pseudoreplication (see “random” parameter from “rma.mv” function of the “metafor” R‐package), testing hypotheses about categorical factors (moderators) and continuous explanatory variables (metaregression), and leading to less biased estimates (with associated confidence interval) than traditional analyses (Viechtbauer, 2010; www.metafor-project.org). Third, we derive specific size effects in order to introduce the LRI‐framework and some elements of the Hunt's (2006, 2012) methods into our synthesis.

Our second goal was to illustrate the potential of our meta‐analysis approach to the study of microevolution, by applying it to a comprehensive literature survey. To this end, we produced the most comprehensive dataset to date of microevolutionary rates in plant quantitative traits.

### 1.1 Haldanes and SMDs

The *haldane* rate was formalized by Gingerich (1993) from Haldane's (1949) original proposal. This rate is defined by Gingerich (2009) and Hendry and Kinnison (1999) as:

(1) $$H=\frac{\frac{\bar{x}{}_{2}-\bar{x}{}_{1}}{SD_{x}}}{\Delta t}$$

where *H* is the *haldane* rate, *x* is the original continuous variable, $\bar{x}{}_{1},\;\bar{x}{}_{2}$ are the sample means of the variable at times 1 and 2, *SD*_*x*_ is the square root of the pooled variance of samples at times 1 and 2, and Δ*t* is the number of generations elapsed between times 1 and 2.

The simplest ES based on mean difference is Cohen's *d* defined as the difference between sample means divided by the pooled sample standard deviation (i.e., $d=\left(\bar{x}{}_{2}-\bar{x}{}_{1}\right)/SD_{x}$). The equivalence between the *haldane*'s numerator and the SMD (Cohen's *d*) is now evident:

(2) $$H\times \Delta t\;=\;\frac{\bar{x}{}_{2}-\bar{x}{}_{1}}{SD_{x}}\;=\;d$$

However, Cohen's *d* is a biased estimator of the actual mean difference which bias increase with small samples (Hedges, 1981). Therefore, it is better to use the SMD Hedges’ *g* (Hedges, 1981), which is an unbiased estimator of the mean difference.

(3) $$g\;=\;d\times J_{m}\;\;\;\;\;;\;\;\;\;\;J_{m}\;=\;1-\frac{3}{4\left(m-2\right)-1}$$

where *m* is the total sample size. When *m* is large enough (e.g., *m *=* *20), the difference between *g* and *d* is negligible.

(4) $$\begin{array}{cc} & d\times J_{\left(m=20\right)}\;=\;d\times 0.96\\ & d\approx g\;\;\Rightarrow \;\;g\approx H\times \Delta t \end{array}$$

When Gingerich (1993) introduced his *haldane* rate for first time, he used ln‐transformed variables. This is because, as the means for morphological traits (used in paleontology) get bigger, the variation also increases; instead, ln‐transformed variables tend to be homocedastic. This has been the most traditional use of the rate, even when transformation is not always necessary. To distinguish the natural log‐transformed definition of *haldane* from the nontransformed one, we use *H* for the latter and *H*_*g*_ for the log‐transformed variable:

(5) $$\begin{array}{cc} & v\;\;=\;\;ln\left(x\right)\\ & H_{g}\;=\;\frac{\frac{\bar{v}{}_{2}-\bar{v}{}_{1}}{SD_{v}}}{\Delta t}\\ & H_{g}\;=\;\frac{\bar{v}{}_{2}-\bar{v}{}_{1}}{SD_{v}\times \Delta t} \end{array}$$

where $\bar{v}{}_{1},\bar{v}{}_{2}$ are the means of the natural logarithm (*ln)* of the original variable (*x*) in times 1 and 2 and *SD*_*v*_ is the standard deviation of the *ln* of the original variable.

(6) $$H_{g}\;\;=\;\;\frac{\bar{v}{}_{2}-\bar{v}{}_{1}}{\Delta t}\times \frac{1}{SD_{v}}$$

However, ecological timescales (from 0 to 300 years or 1 to 10 generations) are very small in comparison with paleontological timescales (from 10² to 10⁷ or more generations and from thousands to hundred millions years; Gingerich, 1983, 2001). It is therefore reasonable to assume that in ecological timescales Δ*t* tends to zero:

(7) $$\begin{array}{cc} \underset{\Delta t\to 0}{\text{lim}}\frac{\bar{v}{}_{2}-\bar{v}{}_{1}}{\Delta t} & =\underset{\Delta t\to 0}{\text{lim}}\frac{ln\left(x_{2}\right)-ln\left(x_{1}\right)}{\Delta t}\\ & =\frac{1}{x}\times \frac{dx}{dt} \end{array}$$

And in discrete time intervals:

(8) $$\frac{1}{x}\;\times \;\frac{dx}{dt}\;\approx \;\frac{1}{\overset{¯}{x}}\;\times \;\frac{\Delta x}{\Delta t}$$

It has been shown (Lewontin, 1966; Lynch, 1990; Wright, 1952) that:

(9) $$SD_{v}\approx {\text{CV}}_{x}\;\;\;\;\;;\;\;{\text{CV}}_{x}\;=\;\frac{SD_{x}}{\overset{¯}{x}}$$

Therefore, the combination of equations (6), (7), (8), (9), in ecological timescales, leads to:

(10) $$\begin{array}{cc} H_{g} & =\frac{\bar{v}{}_{2}-\bar{v}{}_{1}}{\Delta t}\times \frac{1}{SD_{v}}\approx \frac{\Delta x}{\Delta t}\times \frac{1}{\overset{¯}{x}}\times \frac{\overset{¯}{x}}{SD_{x}}\\ & H_{g}=\frac{\bar{v}{}_{2}-\bar{v}{}_{1}}{SD_{v}\times \Delta t}\approx \frac{\bar{x}{}_{2}-\bar{x}{}_{1}}{SD_{x}\times \Delta t} \end{array}$$

(11) $$H_{g}\approx H=\frac{\bar{x}{}_{2}-\bar{x}{}_{1}}{SD_{x}\times \Delta t}$$

Equations (5), (6), (7), (8), (9), (10), (11) show that the shorter the elapsed time, the closer will be *H* and *H*_*g*_. This may be the reason why Hendry and Kinnison (1999) report that the analysis of rates with and without ln‐transformed variables gives similar results. As showed above, *H*'s numerator is the same as the Cohen's *d*; moreover, according to equation (11) we expect that *H*_*g*_'s numerator (which is computed from the *ln* of the original variable) and the SMD to give similar results in the elapsed times considered or under certain conditions of distribution of the variable developed in “SMD and *H*_*g*_'s numerator” section.

### 1.2 Derived effects sizes

In order to incorporate meta‐analysis in the study of microevolutionary rates, it is necessary to have suitable effects sizes, which may be derived from the SMD as explained below. Each ES (as magnitude) is a variable made up by all the outcomes obtained from the literature. Each outcome (as measurement) is an estimation of a realized ES and a measure of the precision of such estimation (its variance). In order to perform a meta‐analysis, we need both the estimators of the amount (and rate) of change and the variances of such estimators.

Because in the study of contemporary evolution the absolute value of the change is often more important than its direction, several authors have analyzed the former (Bone & Farres, 2001; Hendry & Kinnison, 1999; Kinnison & Hendry, 2001). However, SMD is distributed as a noncentral *t*‐variate (Hedges, 1981). Then, the absolute value of such distribution is a folded noncentral *t*‐variate. But also, Hedges (1981) explains that the distribution of the unbiased SMD estimator can be approximated well by a normal distribution when the number of observations or the number of studies (which is our case) is large. As we show below, in our database the log transformation of the unbiased SMD approximates well to a normal distribution. Therefore, it is reasonable to assume that meta‐analysis assumptions are being met with use of the log_10_(|*g*|) (*LG* hereafter). Here we propose a new ES, called *“LG*,*”* and its variance *“V*_*LG*_,*”* calculated according to the propagation errors theory (Bevington & Robinson, 2003):

(12) $$\begin{array}{cc} LG\;=\;log_{10}\left(|g|\right) & \Rightarrow LG\;=\;\frac{ln(|g|)}{ln(10)}\;=\;\frac{1}{ln(10)}\times ln\left(|g|\right)\\ & \Rightarrow \sigma_{LG}\;=\;ln^{-1}\left(10\right)\times \frac{\sigma_{g}}{g}\\ & \Rightarrow \sigma_{LG}^{2}\;=\;ln^{-2}\left(10\right)\times \frac{\sigma_{g}^{2}}{g^{2}} \end{array}$$

(13) $$LG={\text{log}}_{10}\left(\left|g\right|\right)\;\;\;\;;\;\;\;\;V_{LG}\approx 0.1886\times \frac{V_{g}}{g^{2}}$$

We constructed an evolutionary rate which is the ratio between Hedges’ *g* and the time elapsed since the environmental novelty appeared; time may be measured as years or generations. We call this ES “*h*,” bearing in mind that if time is expressed in years, it must not be interpreted as *haldanes*, except in the case of annual species. We call its variance “*V*_*h*_” which may be calculated as the variance of the product of a variable (*g*) by a constant (1/Δ*t*) (if we use “years” as time; Bevington & Robinson, 2003):

(14) $$h=\frac{g}{\Delta t}\;\;\;\;;\;\;\;\;V_{h}=\frac{V_{g}}{{\left(\Delta t\right)}^{2}}$$

If we use “generations” as time, and there is an associated uncertainty which allows the estimation of the variance of “Δ*t*,” then “*V*_h_” is (Bevington & Robinson, 2003):

(15) $$h=\frac{g}{\Delta t}\;\;\;\;;\;\;\;\;V_{h}=\left(\frac{V_{g}}{g^{2}}+\frac{V_{\Delta t}}{{\left(\Delta t\right)}^{2}}-\frac{2\times {\text{Cov}}_{g,\Delta t}}{g\times \Delta t}\right)\times h^{2}$$

Due to the relation between evolutionary rates and elapsed time, already documented by other authors (Gingerich, 1993, 2009; Hendry & Kinnison, 1999), there is a need for a new ES appropriate to the LRI and Hunt framework (Gingerich, 1993, 2009). Here we propose a new ES, called *“LR*,*”* and its variance *“V*_*LR*_,*”* calculated according to the propagation errors theory (Bevington & Robinson, 2003):

(16) $$\begin{array}{cc} LR\;=\;log_{10}\left(\left|h\right|\right) & \Rightarrow LR\;=\;\frac{ln\left(\left|h\right|\right)}{ln\left(10\right)}\;=\;\frac{1}{ln\left(10\right)}\times ln\left(\left|h\right|\right)\\ & \Rightarrow \sigma_{LR}\;=\;ln^{-1}\left(10\right)\times \frac{\sigma_{h}}{h}\\ & \Rightarrow \sigma_{LR}^{2}\;=\;ln^{-2}\left(10\right)\times \frac{\sigma_{h}^{2}}{h^{2}} \end{array}$$

(17) $$LR=log_{10}\left(\left|h\right|\right)\;\;\;\;;\;\;\;\;V_{LR}\approx 0.1886\times \frac{V_{h}}{h^{2}}$$

Here, the concern about the distribution of the proposed ES is also valid. As in the case of the amount of change (*g*) and in the case of rates of change (*h*) the absolute value is more important that the direction. Moreover in order to apply LRI and Hunt's approach, we need to log transform such variables, which is only possible in the case of positive values. As we show below, the distribution of *LR* is also close to a normal distribution.

## 2 MATERIALS AND METHODS

### 2.1 Literature survey

To identify published studies that provide data of changes in quantitative traits of vascular plants in a known temporal framework, we searched Scopus (www.scopus.com), on 22 December 2015, using the following complementary search keys, which were applied to all studies published between 1823 and 2015 (Scopus. Content Coverage Guide, https://www.elsevier.com/solutions/scopus/content):

#### 2.1.1 Search string I

(TITLE‐ABS‐KEY(“rapid *evolution”) OR TITLE‐ABS‐KEY(“contemporary evolution”) OR TITLE‐ABS‐KEY(“rapid divergence”) OR TITLE‐ABS‐KEY(“evolution on ecologica*”) AND TITLE‐ABS‐KEY(plant*) AND NOT TITLE‐ABS‐KEY(gene)) AND SUBJAREA(mult OR agri OR bioc OR immu OR neur OR phar OR mult OR medi OR nurs OR vete OR dent OR heal) AND (LIMIT‐TO(SUBJAREA, “AGRI”) OR LIMIT‐TO(SUBJAREA, “BIOC”) OR LIMIT‐TO(SUBJAREA, “ENVI”) OR LIMIT‐TO(SUBJAREA, “MULT”) OR LIMIT‐TO(SUBJAREA, “EART”) OR LIMIT‐TO(SUBJAREA, “PHAR”)).

#### 2.1.2 Search string II

(TITLE‐ABS‐KEY(*evolution) AND (ALL(darwins) OR ALL(haldanes)) AND TITLE‐ABS‐KEY(plant)) AND SUBJAREA(mult OR agri OR bioc OR immu OR neur OR phar OR mult OR medi OR nurs OR vete OR dent OR heal) AND (LIMIT‐TO(SUBJAREA, “AGRI”) OR LIMIT‐TO(SUBJAREA, “BIOC”) OR LIMIT‐TO(SUBJAREA, “ENVI”) OR LIMIT‐TO(SUBJAREA, “MULT”) OR LIMIT‐TO(SUBJAREA, “EART”) OR LIMIT‐TO(SUBJAREA, “AGRI”) OR LIMIT‐TO(SUBJAREA, “BIOC”) OR LIMIT‐TO(SUBJAREA, “ENVI”) OR LIMIT‐TO(SUBJAREA, “MULT”) OR LIMIT‐TO(SUBJAREA, “EART”)).

#### 2.1.3 Inclusion/exclusion criteria

**Figure 1:**
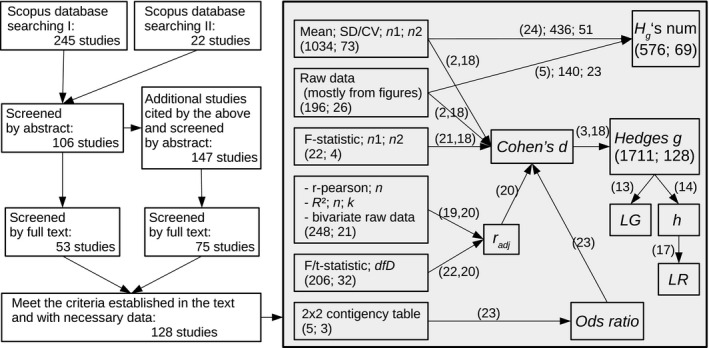
Flow diagram of the literature survey and data extraction. On left side, the sequence of steps in the selection of studies. On the right (grey) side, the type of data source found in papers and steps toward the ES and *H*_g_'s numerator calculation. Numbers in boxes indicate number of outcomes and number of studies, respectively. Number of papers on the right side is not additive because each paper may have different source types. On the arrows, numbers in brackets indicate the equation numbers used in the calculation; the numbers after the brackets, when present, indicate the number of outcomes and studies, respectively

The first survey retrieved 245 studies from 1972 to 2015, and the second showed 22 studies from 2001 to 2011. We also reviewed papers cited by the 267 studies above. We were interested in quantitative trait microevolution processes occurring in “ecological timescales”; therefore, we considered studies that measure intraspecific change in a quantitative trait and report the time elapsed from the onset of the environmental novelty or refer to a historical or biological event reported in other sources (e.g., a mine opening, a well‐documented biological invasion). We included studies which reported situations where the elapsed time between the environmental change and the sampling was no greater than 300 years. The included studies followed a single population through time since a change in the environment, or compared two or more populations, diverging from an originally single population, of vascular plants by measuring a quantitative trait across two situations, where one of them was a new condition of known age (i.e., allochronic and synchronic designs according to Hendry & Kinnison, 1999). As we were interested in phenotypic changes potentially related to environmental changes, we selected studies with plants in natural or experimental conditions but in which reproduction was not manipulated (artificial selection), and which did not include interspecific hybridization, polyploidy, or other chromosomal mutations. All the studies satisfying the conditions above and reporting information allowing the calculation of a standardized mean difference were included in the database. A total of 128 studies were retained for analysis. The environmental changes included expansions of distributional range, exposure to soil or air pollution, herbicides, changes in salinity, pH, climate, or the disturbance or irrigation regime, as well as the addition or loss of species in the local community. All data points were categorized according to moderators (independent variables) detailed in Table 1. Figure 1 illustrates the literature survey (following PRISMA guidelines; Liberati et al., 2009) as well as the computing ES process.

**Table 1:** Moderators used to categorize the data points considered in the meta‐analysis

Moderator	Categories	Description (nonexhaustive)
Publication ID (paperID)	Factor levels: 1–128	Identifier to each publication. All outcomes from the same publication share the paperID value
Specie ID (spID)	Factor levels: 1–152	Identifier to each specie. All outcomes from the same specie share the spID value
Data source type (dt)	Raw data (0)	Available values of the original variable from the two populations (or groups of populations) to be compared
	Descriptive statistics (1)	Mean, *SD*/CV/*SE*, sample sizes of the two populations (or groups of populations) to be compared
	Correlation data (2)	Correlation or determination coefficient, plus sample size, plus number of predictors of the model, or bivariate raw data (mostly extracted from figures)
	Inferential statistics plus degree of freedom (3)	*F*‐ or t‐statistic from a main factor of a linear model, where that factor account for the change explained for the environmental novelty
	Contingency table (4)	Frequency of each category in a contingency table (2 × 2)
	Inferential statistics plus sample sizes (5)	*F*‐statistic from a main factor of a linear model, where that factor account for the change explained for the environmental novelty and available sample sizes
Spacial scale (S)	Local (l)	Comparison between populations separated by less than 10 km
	Regional (r)	Comparison between populations separated by more than 10 km but occurring in the same continent
	Continental (c)	Comparison between populations in different continents
Design (D)	Alochronic (a)	Longitudinal studies, i.e., following a population across time
	Synchronic (s)	Transversal studies, i.e., looking at the divergence between populations in time
Publication year (Y)	None (quantitative)	Year of publication minus year of oldest publication in database (1970)
Elapsed time (t)	None (quantitative)	Time elapsed between the onset of the environmental novelty and the measurement
Environmental change (ec)	Discrete (d)	The environmental change occurs suddenly, and takes place all at once
	Gradual (g)	The environmental change progresses slowly, by degrees
Trait variation source (vs)	Phenotypic (phe)	Study considers the variability present in the field with no possibility to dissect phenotypic plasticity from heritable variability
	Genetic (g)	Transgenerational estudies, i.e., common garden or reciprocal transplant
Growth form (gF)	Nongraminoid herb (herb nongram)	Herbaceous plants with no grasslike appearance, mostly nonmonocotiledoneus
	Graminoid herb (gram)	Herbaceous plants in the Poaceae and other families with a grasslike appearance, all monocotiledoneus
	Woody	Trees, shrubs, woody vines
Life history 1 (l)	Annual (ann)	Natural lifespan up to one year
	Perennial (per)	Natural lifespan longer than two years
	Intermediate (int)	Biennial plants and species described as annuals or short‐living perennials depending on the context
Life history 2 (l2)	Long‐lived (lL)	Perennial (according to life history 1)
	Short‐lived (sL)	Annual and intermediate lifespan (according to life history 1)
Trait type (Tr)	Morphological (m)	Leaf area, specific leaf area (SLA), leaf length and width, leaf shape, leaf number of adult plant, height of adult plant, number of shoots/stems, length of shoots, symmetry, root diameter, root:shoot ratio, root arquitecture, trichome density, size of floral and fruit parts, petiole, and stipules
	Physiological (f)	Photosynthetic and metabolic parameters, tolerance to pollution, salinity, drought, or biomass removal, concentration of several substances in plant tissues
	Individual and population growth parameters (h)	Different ways to express individual growth rate (increase in biomass, height, length, number of leaf or tillers in a elapsed time), increase in number or size of seeds, flowers or fruits, age or size at maturity, seed viability, survival, emergence time of seedlings, offspring dispersal, pollen quantity, and viability
	Biotic relations (r)	Any variable taken as response to a treatment that involves a direct realize biotic relation, such as herbivory, interspecific or intraspecific competition, allelopathy, mycorrhizal or rhizobial colonization, seed or seedlings predation, rhizobial colonization, parasitism
	Phenology (p)	Flowering, fruiting, leafing time
	Phenotypic plasticity (pl)	Considered as a trait in itself, i.e., the change in plasticity independently of the nature of the plastic trait

### 2.2 Calculating ES

When raw data were available (mostly from figures), we computed SMD as in equations (2), (3), and (18); 196 ES (11% of the total) were computed in this way. Also, from these data, we computed *H*_*g*_'s numerator in 140 cases (some variables are not amenable to log transformation). When available, we used means, standard deviations (or standard error or variance), and sample size to compute the unbiased standardized mean difference (Hedges’ *g*), as detailed above, and its variance (*V*_*g*_) as follows (Nakagawa & Cuthill, 2007):

(18) $$V_{g}=V_{d}=\frac{n_{1}+n_{2}}{n_{1}\times n_{2}}+\frac{d^{2}}{2\times \left(n_{1}+n_{2}-2\right)}$$

Out of a total of 1,711 ES, 1,034 (60%) were computed in this way. When these parameters were not available, we used the correlation or determination coefficient (*r* or *R²,* or bivariate raw data mostly from figures) and sample size to compute Cohen's *d* and its variance, as shown below (DeCoster, 2004; Nakagawa & Cuthill, 2007). A total of 248 ES (14.5%) were computed in this way:

(19) $$\begin{array}{cc} & r_{\mathrm{adj}}=\sqrt{1-\frac{\left(n-1\right)\times \left(1-R^{2}\right)}{n-k-1}}\\ & r_{\mathrm{adj}}=\sqrt{R_{\mathrm{adj}}^{2}} \end{array}$$

where *n* is the sample size, *R²* is the determination coefficient of a linear model, *k* is the number of parameters of the model (not including the intercept, if it is the case of a simple regression *k* will be 1), and $R_{\mathrm{adj}}^{2}$ is the adjusted *R²*.

(20) $$d=\frac{2\times r_{\mathrm{adj}}}{\sqrt{1-{r_{\mathrm{adj}}}^{2}}}\;\;\;\;;\;\;\;\;V_{d}=\frac{4\cdot V_{r}}{{\left(1-{r_{\mathrm{adj}}}^{2}\right)}^{3}}\;\;\;\;;\;\;\;\;V_{r}=\frac{{\left(1-{r_{\mathrm{adj}}}^{2}\right)}^{2}}{n-1}$$

Studies reporting r‐Pearson or *R*² in a linear regression measure the change of a quantitative plant trait under a novel environmental condition that manifests itself as a gradient in space or time (a gradient of pollution, precipitation, temperature, etc.). For example, from Antonovics and Bradshaw (1970), we calculated the correlation between different traits reported and the zinc soil concentration in a transect across a mine boundary. From Kollmann and Bañuelos (2004), who studied the evolution of an invasive plant in a new environment, we used the correlation between traits and latitude, which summarize the divergence experienced by the original population in the invasion of new habitats.

When these parameters were not available, we used *F*‐statistic or *t*‐statistic. This included studies comparing two populations, or a population before and after an environmental novelty; such environmental novelty was always the main factor in the analysis and included two levels. For example, Leger and Rice (2007) compared some traits in native (California, USA) and introduced populations (Chile) of *Eschscholzia californica* in a common garden experiment; the main factor explaining the invasion process was country of origin. In cases where F‐statistic and sample sizes were available, we computed Cohen's *d* as follows (DeCoster, 2004):

(21) $$d=\sqrt{\frac{F\times \left(n_{1}+n_{2}\right)}{n_{1}\times n_{2}}}$$

A total of 22 ES (1.3%) were computed in this way. In the cases in which sample size of each group was not available, but the degrees of freedom were, we calculated *r* and its variance (DeCoster, 2004); 206 of the total ES were computed in this way (12%):

(22) $$r=\sqrt{\frac{F}{F+dfD}}\;\;\;\;;\;\;\;\;r=\frac{\sqrt{t^{2}}}{t^{2}+dfD}$$

where *dfD* are the degrees of freedom of the denominator in the calculation of the statistic. Finally, when no other information was available, we used 2 × 2 tables of frequency to calculate Cohen's *d* from odds ratio (*OdR*) as follows:

(23) $$\begin{array}{cc} & OdR=\frac{n_{1}{}_{T}\times n_{0}{}_{C}}{n_{0}{}_{T}\times n_{1}{}_{C}}\;\;\;\;;\;\;\;\;V_{ln\left(OdR\right)}=\frac{1}{n_{1}{}_{T}}+\frac{1}{n_{0}{}_{T}}+\frac{1}{n_{1}{}_{C}}+\frac{1}{n_{0}{}_{C}}\\ & d=ln\left(OdR\right)\times \frac{\sqrt{3}}{\pi }\;\;\;\;;\;\;\;\;V_{d}=V_{ln\left(OdR\right)}\times \frac{3}{\pi^{2}} \end{array}$$

where *ln(OdR)* is the natural logarithm of the odds ratio; *n* is the absolute frequency specified in the subindex; “1T”: positives in the treatment; “0T”: negatives in the treatment; “1C”: positives in the control; “0C”: negatives in the control. Five ES (0.3%) were computed in this. The odds ratio appears in some studies where authors measured a binomial variable (e.g., germination, presence of roots, survival) in an experiment with plants coming from two sources; this generated the 2 × 2 contingency table. For example, Wu and Bradshaw (1972) measured tolerance to copper in plant populations near an industrial source and populations from noncontaminated sites. Tillers from each site were suspended in a copper or control solution; the response variable was the subsequent development (yes/no) of roots. This provided a metric of the copper tolerance of each population.

From Cohen's *d*, we computed Hedges’ *g* (equation (3)), and finally, Hedges’ *g* was transformed to absolute value, because the direction of change does not concern us, only its amount. Then, from the Hedges’ *g*, we calculated the derived ES *LG, h*, and *LR* (equations (13), (14), (17)). When necessary, data were extracted from figures using Engauge Digitizer 4.1 software (Mitchell, 2002). In order to explore possible bias introduced by these different sources of information, we analyzed *LG* moderated by source type in a mixed model with random factor paperID (see Table 1 for moderator details).

Trim and fill procedure (Duval & Tweedie, 2000) for exploring the publication bias of the database was not applicable. This is because of the procedure assumes a symmetrical distribution. However the expected distribution of a random collection of amounts and rates of change is a normal distribution with mean equal to zero; therefore, its absolute value is a folded normal distribution (Tsagris, Beneki, & Hassani, 2014). As consequence, the log transformation of such folded normal distribution is left‐skewed (not symmetrical).

### 2.3 SMD and *H*_*g*_'s numerator

To determine whether SMD is a good estimator of the *H*_*g*_ numerator, and under which conditions, we use empirical data from the literature survey and simulated data. With empirical data, we evaluated the similarity of the two metrics (*H*_*g*_'s numerator and SMD). According to Lynch (1990), *H*_*g*_'s numerator can be estimated using the equation (24), if the CVs are <0.3:

(24) $$H_{g}\times \Delta t=\frac{\left(ln\left(\bar{x}{}_{2}\right)-0.5\;\text{CV}{\;}_{\left(x{}_{2}\right)}^{2}\right)-\left(ln\left(\bar{x}{}_{1}\right)-0.5\;\text{CV}{\;}_{\left(x{}_{1}\right)}^{2}\right)}{{\text{CV}}_{x}}$$

where $\bar{x}{}_{1}$ is mean of population 1, $\bar{x}{}_{2}$ is mean of population 2, $CV_{x_{2}}$ is the variation coefficient of population 2, $CV_{x_{1}}$ is the variation coefficient of population 1, and CV_*x*_ is the variation coefficient of total population calculated from pooled variance and global mean.

In this way, for the cases in which raw data were not available, we computed *H*_*g*_'s numerator, whenever possible, by using expression (16) and compared it, by correlation and linear regression, with the SMDs (Cohen's *d* and Hedges’ *g*) computed from the same cases.

It is not possible to know, from the analysis of these empirical data, how some aspects of the distribution of the original variable affected the correlation between *H*_*g*_'s numerator and SMD. These aspects include the relation between mean and *SD*, specifically whether or not the CV holds constant when mean and *SD* vary. We therefore generated two sets of simulated data with different mean–*SD* relations. We compared random pairs of samples. For each comparison, *H*_*g*_'s numerator and SMD were computed, and the correspondence between them was analyzed by simple correlation (Figure 3). The samples to be compared had 10 elements each, from a random normal distribution. In the first set of simulated data, the *SD* of each sample was set to be proportional to the sample mean, so that the expected CV is held constant. In the second set of simulations, the *SD* of each sample was set to be independent from its mean and came from a random normal distribution. In both sets of simulations, we covered the range of maximum CV between 3 and 0.01. Each set of simulations included 1,000 iterations. In each of these 1,000 iterations, 10,000 comparisons between samples were performed. Because the calculation of *H*_*g*_'s numerator requires a positive values variable, we set up the simulations so that negative values were rare (no negative values were found in the fist set of simulated data, and only 5.5% of the total in the second one); when negative values did appear, we substituted them by 0.001.

### 2.4 LRI‐framework, the Hunt approach, and ES

Gingerich (2009) showed the use of LRI by mean of a series of simulations. To test the utility of the ES *LR* in analyzing microevolutionary rates according to the LRI‐framework, we performed a simulation developed by Gingerich (2009). The simulation reproduced here is a random walk temporal series. In the simulation, we started with a sample of 30 elements with mean = 100 and variance = 1. In each generation (*t*), until *t* = 200, we sampled 30 elements from a mean equal to mean in the previous time (*t*−1) plus a random deviation (from a normal distribution, mean = 0 and *SD* = 1.25) and variance = 1. We then established all the possible comparisons from each series and computed *H*_*g*_ and *LR*. Then we constructed a linear regression of log_10_(*H*_*g*_) as a function of log_10_(time) and a metaregression of *LR* as a function of log_10_(time). To do this, we used the function “rma.uni” from the “metafor” R‐package to perform a random effect model, method REML, weighted by the inverse of variance. Due to limited computing capacity, the metaregression was performed with a random subset of 5,000 comparisons (from a total of 20,100 comparisons). The simple linear regression was performed with the same subset than the metaregression to compare the two methods. This procedure was performed to show that metaregression is equivalent to the LRI if all samples have similar weights (due to equal sample sizes). From data of the same simulation, we computed *LG*, and from the same subset we performed a metaregression of *LG* as a function of log_10_(time) to illustrate the applicability of the *LG* ES in the graphical tool described by Hunt.

All simulations and statistical analyses were made in R (R Core Team, 2015). Scripts are provided as supplementary material.

## 3 RESULTS

### 3.1 Summary of results of the literature survey

**Figure 2:**
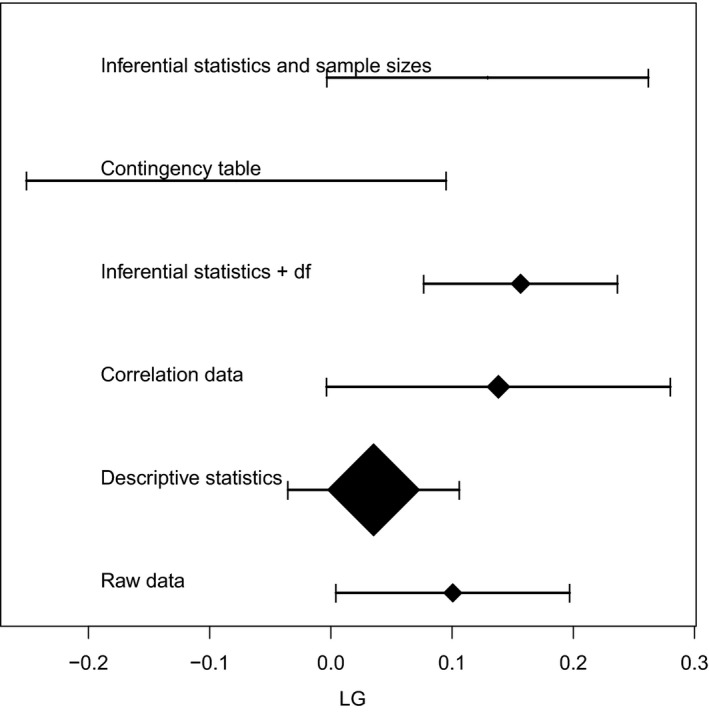
Forest plot of the overall *LG* ES for each data source type. Segments represent the 95% confidence interval of the overall *LG* ES for each source type, and the central symbol in each segment represents the mean. Symbol sizes are proportional to the number of outcomes summarized by each source type. On the left, the name of each source type according to Table 1

Through a systematic search and application of meta‐analysis techniques, we were able to obtain 1,711 ES, many more times than what had been previously possible by calculating evolutionary rates (22 to 8 more outcomes, and 11 to 6 more species). These 1,711 ES correspond to the change in populations of 152 species of plants, from 34 families, in an elapsed time of <260 year and covering a wide range of traits, habits, and environmental situations. With previous metrics, we could have only incorporated cases in which we could compute *H*_*g*_'s numerator, only 576 cases in the dataset, from 66 species, belonging to 20 families. Although the number of publications on microevolution has grown sharply in the last 45 years (Figure S1), extremely few of them use standardized metrics (e.g., only one paper in our dataset, Crispo et al., 2010; reports in *haldanes)*, rendering our approach particularly useful. Additionally, we show that different sources of information do not introduce bias (Figure 2), only the ES computed from contingency tables tends to be different, but there are only five outcomes of this type; therefore, the sample error is expected to be high.

The overall pattern of amount (*LG*) and rates of change (*LR*) as a function of the elapsed time is shown in Figures S4 and S5, respectively. The distribution of *LG* and *LR* ES was close to normal (Figures S2 and S3), which respects the assumptions of meta‐analysis. At the same time, this suggests a publication bias toward high amounts and rates of change. As pointed above, if the original distribution of amounts and rates of change is a centered normal distribution, the log‐transformed of the absolute values of such distribution is left‐skewed, so that the observed symmetry may be due to a higher than expected frequency of extreme values.

### 3.2 SMD and *H*_*g*_'s numerator

We had raw data for 196 cases, from which 140 allow to calculate *H*_*g*_'s numerator. Additionally, we had enough information to apply equation (24) to 1,034 cases (out of 1,711). Of these, only 436 met the requirement (CV < 0.3) to apply such equation (Lynch, 1990; Hendry & Kinisson 1999). All these 576 cases were used to test the correspondence between *H*_*g*_'s numerator and SMD with real data. This dataset covers a CV range from 0.0147 to 1.79. The correlations between *H*_*g*_'s numerator and SMDs showed values near 0.98 and were highly significant in both cases (*H*_g_*t vs. Hedges’ *g*:* r* = 0.9797, *p *< 0.0001; *H*_g_*t vs. Cohen's *d*:* r* = 0.9825, *p *< 0.0001). The linear regression showed a tight fit between *H*_*g*_'s numerator and SMDs (Table 2). Hedges’ *g* slightly underestimated the amount of change, whereas Cohen's *d* overestimated it (Table 2). We also found that the relative difference between SMD and *H*_*g*_'s numerator (i.e., (*H*_*g*_'s numerator – SMD)/*H*_*g*_'s numerator) increases with the CV of the original variable (Figure S6). Based on our data, we were unable to test the correspondence between SMD, computed from other ES (such as *OdsRatio* or *r*), and *H*_*g*_'s numerator, but we used equivalences previously tested in the literature (DeCoster, 2004).

**Table 2:** Correspondence between Hg's numerator and SMD in real data

*H*_*g*_'s numerator = β_0_ + β_1_*SMD + Er				
SMD	β_0_ [CI 95%]	β_1_ [CI 95%]	*R*²	*p*‐value
Cohen's *d*	0.02 (−0.02; 0.07)	0.97 (0.95; 0.98)	0.96	<0.0001
Hedges’ *g*	−0.03 (−0.08; 0.02)	1.10 (1.08; 1.12)	0.96	<0.0001

**Figure 3:**
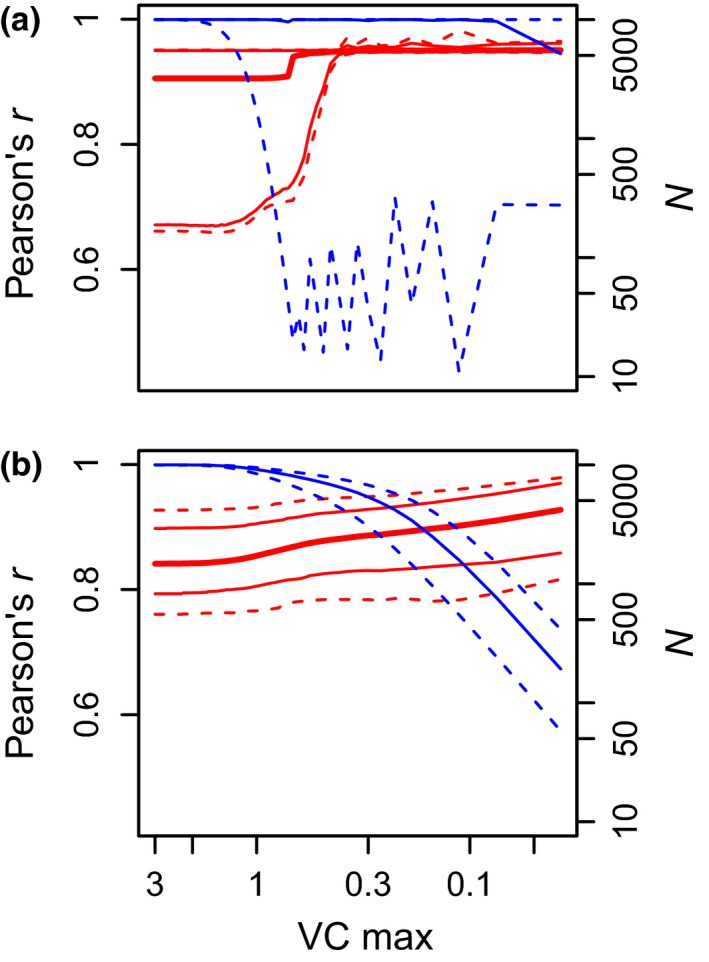
Pearson's correlation coefficient between Hedges’ *g* and *H*_*g*_'s numerator as a function of maximum CV of the sample. Solid thick red line: median of Pearson's correlation coefficient (*r*) across 1,000 simulations; solid thin red line: 0.1 and 0.9 quantiles of Pearson's correlation coefficient (*r*) across 1,000 simulations; dashed red line: 0.05 and 0.95 quantiles of Pearson's correlation coefficient (*r*) across 1,000 simulations; blue solid and dashed lines indicate the median and 0.05–0.95 quantiles for the sample size (N) at each level of maximum CV, respectively. Panel (a): simulation series with expected CV constant; panel (b): simulation series with *SD* independent of sample mean

Using a simulated dataset, we found that SMD was always significantly and positively correlated with *H*_*g*_'s numerator, the correlation coefficient rising when CVs in both samples decrease. The Pearson's correlation coefficient is, on average, >0.8 through the whole range of maximum CV (0.01–3; Figure 3).

### 3.3 LRI‐framework, Hunt approach, and ES

**Figure 4:**
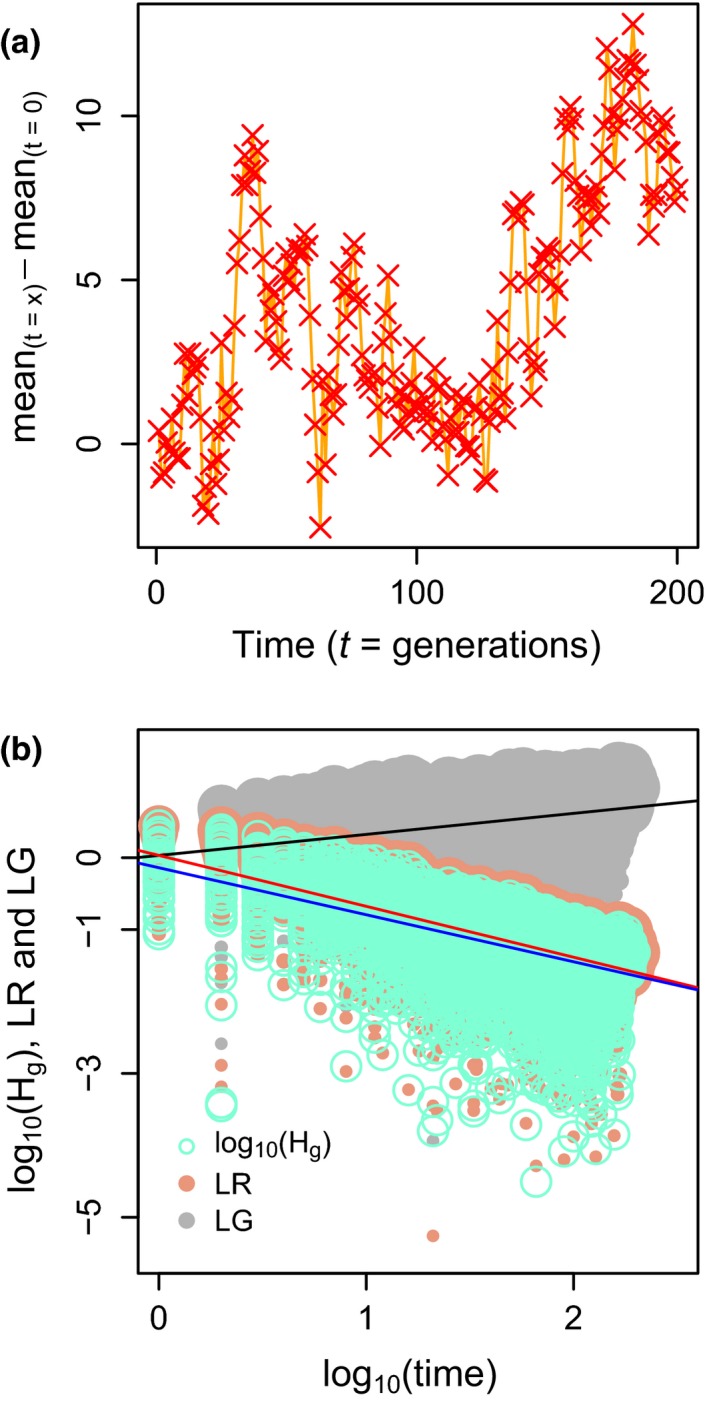
Simulated random walk and its representation in the LRI‐framework. Panel (a): change in the mean of the trait in the random walk. Panel (b): all possible comparisons between points in the random walk and adjusted regression line in pink color are the *LR* points, whereas in light blue are log_10_(*H*_g_) points; the red and blue straight lines show the adjusted metaregression and simple linear regression of *LR* and log_10_(*H*_g_) as a function of log_10_(time), respectively. Grey points represent *LG* ES, and the black line shows the metaregression of *LG* and log_10_(time). Symbol sizes in panel (b) represent the relative weight in the analysis

In order to compare *LR* with the LRI‐framework and illustrate the applicability of a tool derived from the Hunt's methods (Hunt, 2006, 2012), we used a random walk temporal series. Figure 4 shows the results from a particular random series, which is close to the average expected for this kind of series according to Gingerich (2009; fractal dimension *D* = 1.5), and in which we analyzed the linear regression for the log_10_(*H*_*g*_) and the metaregression for *LR* and *LG* ES. Similar (but significantly different) slopes were estimated by LRI and the metaregression (*LR*), whereas the intercept of the LRI was lower (Table 3). As predicted by Hunt, in an unbiased random walk, the slope of the rate of change (*LR*) with elapsed time was negative, whereas the slope of the amount of change (*LG*) with elapsed time was positive (Table 3).

**Table 3:** Application of the LRI‐framework by standard linear model or by metaregression with the dataset of the random path shown in Figure 4a and metaregression of the *LG* EF

*Y *= β_0_ + β_1_*log_10_(time) + Er		
Y [*n*]	β_0_ [95% CI]	β_1_ [95% CI]
log_10_(*H*_*g*_) [5000]	−0.139 [−0.191; −0.088]	−0.654 [−0.683; −0.624]
*LR* [5000]	0.032 [−0.005; 0.069]	−0.708 [−0.729; −0.686]
*LG* [5000]	0.032 [−0.005; 0.069]	0.292 [0.271; 0.313]

## 4 DISCUSSION

Patterns of phenotypic evolution in quantitative traits provide useful insight into evolutionary processes. A proper estimation of these patterns requires as much as possible empirical data, expressed in a standardized way. Here we show how that meta‐analysis tools may be used to build a much broader database than it is possible by means of previous techniques. We also provide an ES (*h*), with its variance (*V*_h_), analog to the previous standard metrics used to analyze evolutionary rates (*H* and *H*_*g*_). Finally, we provide suitable ES (*LG* and *LR*) applicable to different analyses like Gingerich's (2009) the LRI‐framework and (a reduced form of) the Hunt's approach (Hunt, 2006, 2012). In both cases, our two available measures (*LG* and *LR*) do not discount the sample error but account for sampling error by weighing the observation according to its precision. These ES contain similar information to that of previous metrics, with the added advantage of being able to use more diverse data (through the application of meta‐analysis), and taking into account methodological concerns, pseudoreplication and “representativity” of the studies issues (Crispo et al., 2010) about this kind of synthesis. All these features allow the application of preexisting conceptual frameworks (Gingerich, 1983; Hendry & Kinnison, 1999; Hunt, 2006, 2012) to the analysis of a much broader database.

As shown in Table 2 and Figure 3, SMDs can be compared with or be used as estimator of *H*_*g*_'s numerator. Although *H*'s numerator and Cohen's *d* are equivalent (equation (2)), it is advisable to use Hedges’ *g* (instead of Cohen's *d*) to avoid possible bias introduced by studies with small sample sizes. Usually, microevolution studies sample a large number of individuals, but as the scale of analysis broadens, the units of observation are populations, and the sample size thus gets smaller. In studies of range expansion, or “invasions,” authors often measure some traits in a number of populations in the original and “invaded” ranges. In these studies of continental or global resolution, the samples are populations rather than individuals (and the available data tend to be trait values of each population). Furthermore, the use of ln‐transformation as suggested by Gingerich is not mandatory. It is recommended for some traits, especially morphological ones, in which *SD* is expected to increase with the mean (CV remains relative constant), in order to reduce heteroscedasticity (Hendry & Kinnison, 1999). Otherwise, the use of raw variable is preferred. Only when CV remains constant, the use of SMDs without transforming the data causes an underestimation of the amount of change. This is because, if CV remains constant, the pooled variance of the raw data is larger than the pooled variance of the *ln‐*transformed variable, so that the amount of change relative to *SD* is smaller.

Considering that the analysis of the random walk temporal series by the *LR* ES and LRI‐framework showed consistent result (Table 3 and Figure 4b), these methods could be both considered valid alternatives. However, metaregression has the advantage of weighting each observation (for estimator variance or sample size) in order to obtain a better parameter estimation. In the current simulation, all samples have the same size, so all comparisons have similar weight in the metaregression and, as expected, the results are similar. This is because we performed the simulation to show the similarity between the two methods. However, in the analysis of real data there will be different sample sizes and variances, and therefore, this weighting becomes relevant for better estimation of parameters and more precise confidence intervals. The possibility of incorporating other moderator variables which can explain the heterogeneity of the data is also an advantage. These strengths of the meta‐analysis become especially relevant when observations from independent temporal series, with different sample sizes, different variances, and the influence of other methodological or biological factors, are all combined in a single analysis.

While performing similarly, meta‐analysis tools present a number of advantages over the conventional calculation of phenotypic and evolutionary changes. Meta‐analysis provides procedures to deal with heterogeneous data sources, investigate the effect of several variables (moderators), and avoid bias due to unrepresentative studies, by the use of the correction factor in Hedges’ *g* and weighting of each ES according to its precision in the analysis. The resulting database and proposed methodology could be useful in addressing relevant questions regarding ecology and evolution, with theoretical and practical implications. Questions that could find more robust answers by their implementation include, e.g., those about the relation between microevolutionary change and elapsed time, the mode of evolution underling observed patterns, the change that plants may experience in a certain time window, and effects of the growth form, life history, and trait type on the tempo and mode of plant microevolution. In summary, the meta‐analysis technique is a powerful tool in addressing questions about microevolution that requires the synthesis of large bodies of empirical data.

## CONFLICT OF INTEREST

None declared.

## References

[ece33116-bib-0001] Alexander, T. J. , Vonlanthen, P. , & Seehausen, O. (2016). Does eutrophication‐driven evolution change aquatic ecosystems? Philosophical Transactions of the Royal Society B: Biological Sciences, 372(1712), 20160041. 10.1098/rstb.2016.0041 PMC5182437 27920386

[ece33116-bib-0002] Antonovics, J. , & Bradshaw, A. D. (1970). Evolution in closely adjacent plant populations. VIII. Clinal patterns at mine boundary. Heredity, 25, 349–362. 10.1038/sj.hdy.6800835 16639420

[ece33116-bib-0003] Bassar, R. D. , Marshall, M. C. , López‐Sepulcre, A. , Zandonà, E. , Auer, S. K. , Travis, J. , … Reznick, D. N. (2010). Local adaptation in Trinidadian guppies alters ecosystem processes. Proceedings of the National Academy of Sciences of the United States of America, 107(8), 3616–3621. 20133670 10.1073/pnas.0908023107 PMC2840427

[ece33116-bib-0004] Bevington, P. R. , & Robinson, D. K. (2003) Chapter 3, Error analysis In Bruflodt D. & Cotkin S. J. (Eds.), Data reduction and error analysis for the physical sciences. 3rd ed. (pp. 36–49). New York: McGraw‐Hill Higer Education.

[ece33116-bib-0005] Bone, E. , & Farres, A. (2001). Trends and rates of microevolution in plants. Genetica, 112–113(1), 165–182. 11838764

[ece33116-bib-0006] Bookstein, F. L. (1987). Random walk and the existence of evolutionary rates. Paleontological Society, 13(4), 446–464.

[ece33116-bib-0007] Carroll, S. P. , Hendry, A. P. , Reznick, D. N. , & Fox, C. W. (2007). Evolution on ecological time‐scales. Functional Ecology, 21(3), 387–393.

[ece33116-bib-0008] Crispo, E. , Dibattista, J. D. , Correa, C. , Thibert‐plante, X. , Mckellar, A. E. , Schwartz, A. K. , … Hendry, A. P. (2010). The evolution of phenotypic plasticity in response to anthropogenic disturbance. Evolutionary Ecology Research, 12(1), 47–66.

[ece33116-bib-0009] Darimont, C. T. , Carlson, S. M. , Kinnison, M. T. , Paquet, P. C. , Reimchen, T. E. , & Wilmers, C. C. (2009). Human predators outpace other agents of trait change in the wild. Proceedings of the National Academy of Sciences of the United States of America, 106(3), 952–954. 19139415 10.1073/pnas.0809235106 PMC2630061

[ece33116-bib-0010] DeCoster, J. (2004) ‘Meta‐Analysis Notes’, Retrieved from http://www.stat-help.com/notes.html 12‐21‐2013.

[ece33116-bib-0011] Duval, S. , & Tweedie, R. (2000). Trim and Fill: A simple funnel‐plot‐based method of testing and adjusting for publication bias in meta‐analysis. Biometrics, 56(2), 455–463. 10877304 10.1111/j.0006-341x.2000.00455.x

[ece33116-bib-0012] Gingerich, P. D. (1983). Rates of evolution: Effects of time and temporal scaling. Science, 222(4620), 159–161. 17741657 10.1126/science.222.4620.159

[ece33116-bib-0013] Gingerich, P. D. (1993). Quantification and comparison of evolutionary rates. American Journal of Science, 293‐A, 453–478.

[ece33116-bib-0014] Gingerich, P. D. (2001). Rates of evolution on the time scale of the evolutionary process. Genetica, 112–113(1), 127–144. 11838762

[ece33116-bib-0015] Gingerich, P. D. (2009). Rates of evolution. Annual Review of Ecology, Evolution, and Systematics, 40(1), 657–675.

[ece33116-bib-0016] Gurevitch, J. , Curtis, P. S. , & Jones, M. H. (2001). Meta‐analysis in ecology. Advances in Ecological Research, 32, 199–247.

[ece33116-bib-0017] Hairston, N. G. , Ellner, S. P. , Geber, M. A. , Yoshida, T. , & Fox, J. A. (2005). Rapid evolution and the convergence of ecological and evolutionary time. Ecology Letters, 8(10), 1114–1127.

[ece33116-bib-0018] Haldane, J. B. S. (1949). Suggestion as to quantitative measurement of rates of evolution. Evolution, 3(1), 51–56. 18115117 10.1111/j.1558-5646.1949.tb00004.x

[ece33116-bib-0019] Hedges, L. V. (1981). Distribution theory for Glass's estimator of effect size and related estimators. Journal of Educational Statistics, 6(2), 107–128.

[ece33116-bib-0020] Hedges, L. V. , Gurevitch, J. , & Curtis, P. S. (1999). The meta‐analysis of response ratios in experimental ecology. Ecology, 80(4), 1150–1156.

[ece33116-bib-0021] Hendry, A. P. (2016). Eco‐evolutionary dynamics. Princeton, NJ: Princeton University Press.

[ece33116-bib-0022] Hendry, A. P. , & Kinnison, M. T. (1999). The pace of modern life, Measuring rates of contemporary microevolution. Evolution, 53(6), 1637–1653. 28565449 10.1111/j.1558-5646.1999.tb04550.x

[ece33116-bib-0023] Hunt, G. (2006). Fitting and comparing models of phyletic evolution: Random walks and beyond. Paleobiology, 32(4), 578–601.

[ece33116-bib-0024] Hunt, G. (2012). Measuring rates of phenotypic evolution and the inseparability of tempo and mode. Paleobiology, 38(3), 351–373.

[ece33116-bib-0025] Kinnison, M. , & Hendry, A. P. (2001). The pace of modern life II: From rates of contemporary microevolution to pattern and process. Genetica, 112–113(1), 145–164. 11838763

[ece33116-bib-0026] Kollmann, J. , & Bañuelos, M. J. (2004). Latitudinal trends in growth and phenology of the invasive alien plant Impatiens glandulifera (Balsaminaceae). Diversity and Distributions, 10, 377–385.

[ece33116-bib-0027] Leger, E. A. , & Rice, K. J. (2007). Assessing the speed and predictability of local adaptation in invasive California poppies (*Eschscholzia californica*). Journal of Evolutionary Biology, 20(3), 1090–1103. 17465919 10.1111/j.1420-9101.2006.01292.x

[ece33116-bib-0028] Lewontin, R. C. (1966). On the measurement of relative Variability. Systematic Zoology, 15(2), 141–142.

[ece33116-bib-0029] Liberati, A. , Altman, D. G. , Tetzlaff, J. , Mulrow, C. , Gøtzsche, P. C. , Ioannidis, J. P. A. , … Moher, D. (2009). The PRISMA statement for reporting systematic reviews and meta‐analyses of studies that evaluate health care interventions: Explanation and elaboration. Plos Medicine, 6(7), e1000100. 19621070 10.1371/journal.pmed.1000100 PMC2707010

[ece33116-bib-0030] Lynch, M. (1990). The rate of morphological evolution in mammals from the standpoint of the neutral expectation. American Naturalist, 136(6), 727–741.

[ece33116-bib-0031] Merilä, J. , & Hendry, A. P. (2014). Climate change, adaptation, and phenotypic plasticity: The problem and the evidence. Evolutionary Applications, 7(1), 1–14. 24454544 10.1111/eva.12137 PMC3894893

[ece33116-bib-0032] Mitchell, M. (2002). Engauge Digitizer. A free open‐source software to extract data points from a graph image, SourceForge. Retrieved from http://digitizer.sourceforge.net

[ece33116-bib-0033] Nakagawa, S. , & Cuthill, I. C. (2007). Effect size, confidence interval and statistical significance: A practical guide for biologists. Biological Reviews, 82(4), 591–605. 17944619 10.1111/j.1469-185X.2007.00027.x

[ece33116-bib-0034] R Core Team (2015). R: A language and environment for statistical computing. R Foundation for Statistical Computing Vienna, Austria: Retrieved from https://www.R-project.org

[ece33116-bib-0035] Sheets, H. D. , & Mitchell, C. E. (2001). Why the null matters: Statistical tests, random walks and evolution. Genetica, 112–113, 105–125. 11838761

[ece33116-bib-0036] Shefferson, R. P. , & Salguero‐Gómez, R. (2015). Eco‐evolutionary dynamics in plants: Interactive processes at overlapping time‐scales and their implications. Journal of Ecology, 103(4), 789–797.

[ece33116-bib-0037] Tsagris, M. , Beneki, C. , & Hassani, H. (2014). On the folded normal distribution. Mathematics, 2, 12–28.

[ece33116-bib-0038] Viechtbauer, W. (2010). Conducting meta‐analyses in R with the metafor package. Journal of Statistical Software, 36(3), 1–48.

[ece33116-bib-0039] Westley, P. A. H. (2011). What invasive species reveal about the rate and form of contemporary phenotypic change in nature. The American Naturalist, 177(4), 469–509. 10.1086/658902 21460571

[ece33116-bib-0040] Wright, S. (1952). ‘The genetics of quantitative variability’, in Quantitative inheritance. Papers read at a colloquium held at the Institute of Animal Genetics Edinburgh University under the auspices of the Agricultural Research Council April 4th to 6th, 1950, pp. 5–41.

[ece33116-bib-0041] Wu, L. , & Bradshaw, A. D. (1972). Aerial pollution and the rapid evolution of copper tolerance. Nature, 238(5360), 167–169.

[ece33116-bib-0042] www.metafor-project.org Last modified: 2016/09/12 20:50 by Wolfgang Viechtbauer

