# Supplementary material for: A novel meta‐analytical approach to improve systematic review of rates and patterns of microevolution
Source: Ecol Evol. 2017 Jun 20;7(15):5821–32. doi: 10.1002/ece3.3116 (PMC5551081; doi:10.1002/ece3.3116)
Supplement: Supplementary file 1 [file ECE3-7-5821-s001.docx]

**Supporting information**


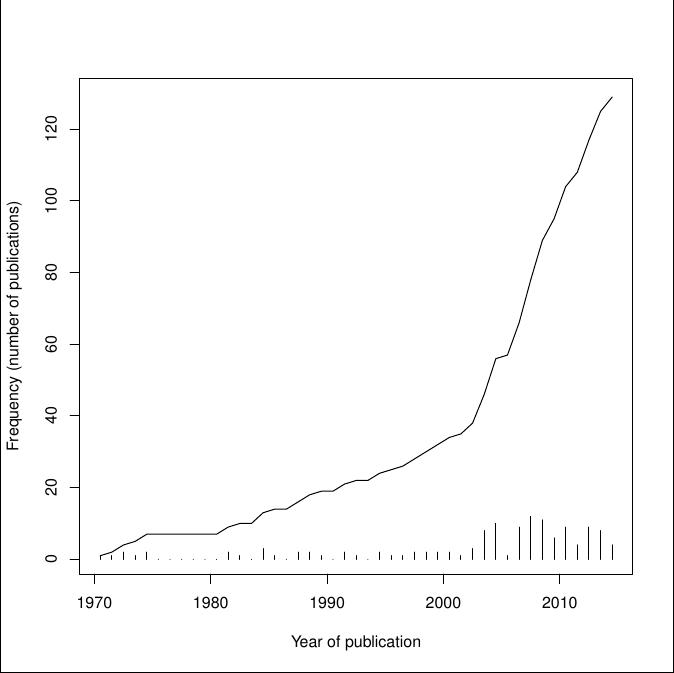


Supp. Fig. 1. Frequency distribution of publications in our database over time. Vertical lines represent the number of publication in each year. Continuous curve shows the cumulative frequency across years.


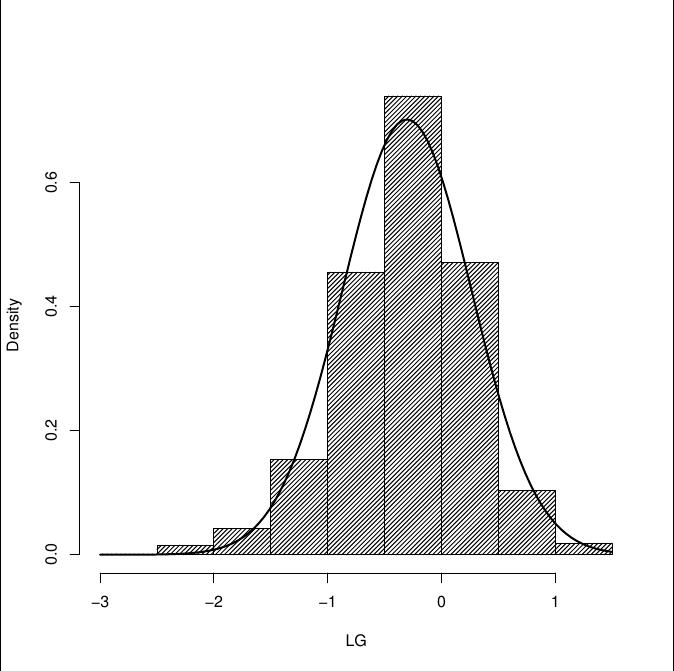


Supp. Fig. 2. Histogram of the *LG* ES. The curve show the normal density function with mean and SD equal to mean and SD of the *LG* distribution.


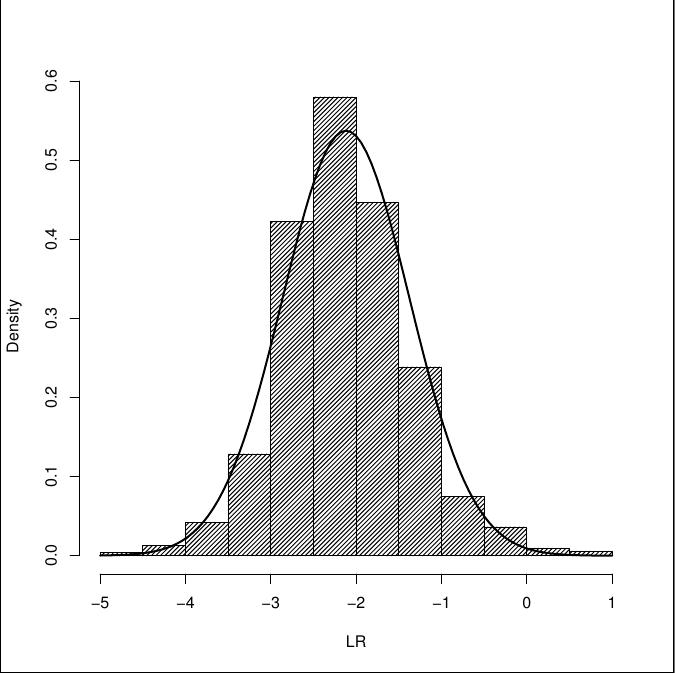


Supp. Fig. 3. Histogram of the *LR* ES. The curve show the normal density function with mean and SD equal to mean and SD of the *LR* distribution.


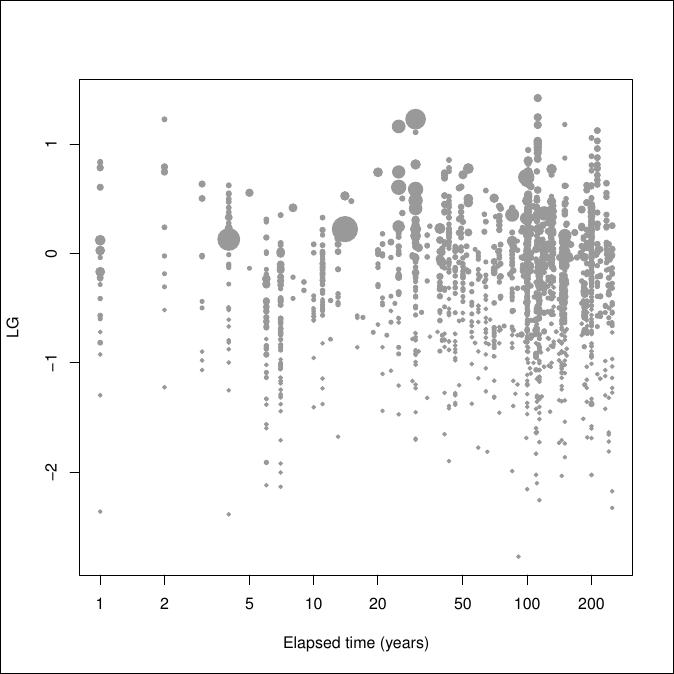


Supp. Fig. 4. LG as function of the log_10_(years). The symbol size is proportional to the weight of each outcome.


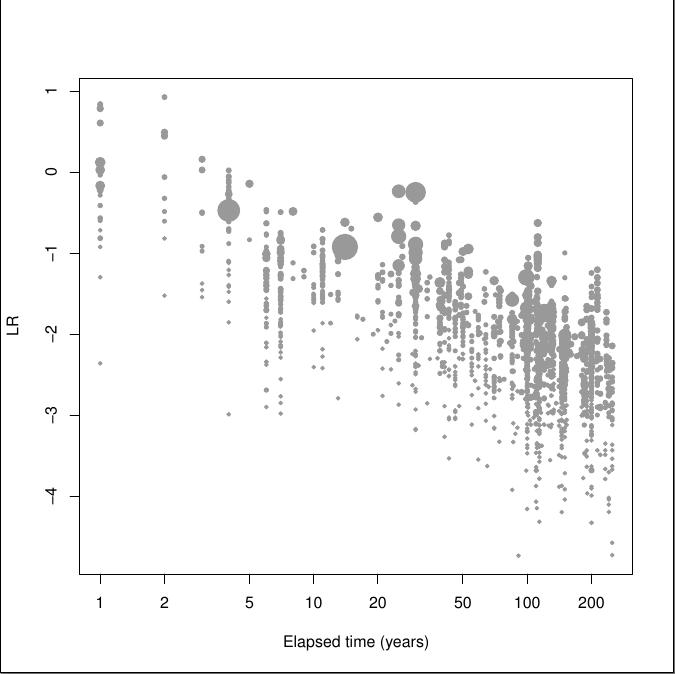


Supp. Fig. 5. LR as function of the log_10_(years). The symbol size is proportional to the weight of each outcome.


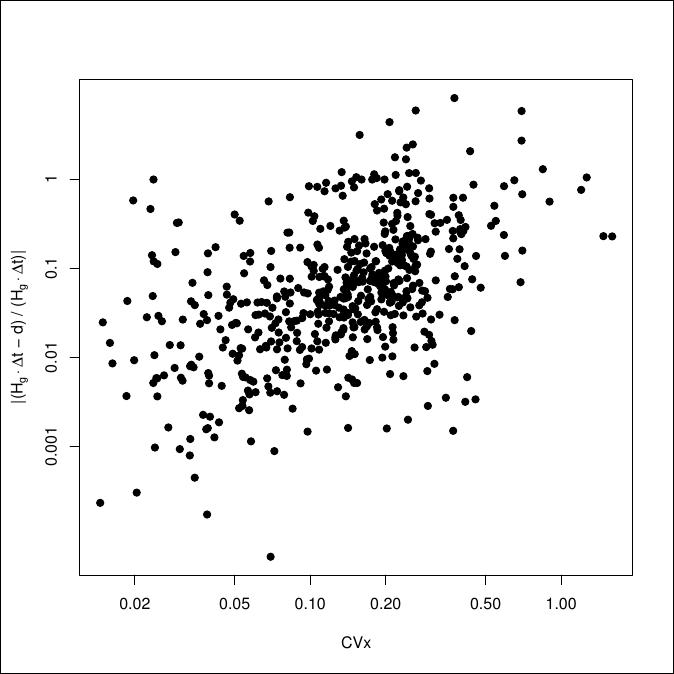


Supp. Fig. 6. Relative absolute difference between SMD (Cohen’s *d*) and *H_g_*’s numerator as function of the CV of the original variable. Both axis are in log scale.

SMD and *H_g_*'s numerator

The code below performs two sets of simulations with different mean-standard deviation (SD) relations. In the first set the SD of each sample is proportional to the sample mean, so that the expected variation coefficient (CV) is held constant (option 1). We use a series of 10 expected CV to cover the full range of maximum CV between 3 and 0.01. In the second set of simulations, the SD of each sample is independent from its mean and comes from a random normal distribution with mean and SD equal to a fixed parameter (option 2). In this case we used four expected SD values to cover the range of maximum CV between 3 and 0.01. Each set of simulations sum 1000 iterations. In each of these 1000 iterations 10,000 comparisons between samples were performed. The samples to be compared had 10 elements each, from a random normal distribution. Negative values were substituted by 0.001. *H_g_*'s numerator and SMD for each comparison was computed.

option <- 1 #choice between 1 and 2

a <- c(2, 1.5, 1, 0.7, 0.5, 0.3, 0.2, 0.1, 0.05, 0.01)# expected value of CV (option 1)

n <- 10

obs <- 10000

h <- 100

s <- 1000 #repetitions

div <- 80

nn <- matrix(nrow=div, ncol=s)#N° of observations in each division

r <- matrix(nrow=div, ncol=s)

CVmax <- matrix(nrow=div, ncol=s)#upper limit of r in each simulation

set.seed(20)

j=1

for (j in 1:s){

mediax <- runif(2*obs,1,h)

#option 1.

if(option==1){

if(j <= 10*s/10){sdx <- a[10]*mediax}

if(j <= 9*s/10){sdx <- a[9]*mediax}

if(j <= 8*s/10){sdx <- a[8]*mediax}

if(j <= 7*s/10){sdx <- a[7]*mediax}

if(j <= 6*s/10){sdx <- a[6]*mediax}

if(j <= 5*s/10){sdx <- a[5]*mediax}

if(j <= 4*s/10){sdx <- a[4]*mediax}

if(j <= 3*s/10){sdx <- a[3]*mediax}

if(j <= 2*s/10){sdx <- a[2]*mediax}

if(j <= 1*s/10){sdx <- a[1]*mediax}

}

#option 2.

if(option==2){

if(j <= 4*s/4){sdx <- abs(rnorm(2*obs, mean=h/8, sd=h/8))}

if(j <= 3*s/4){sdx <- abs(rnorm(2*obs, mean=h/12, sd=h/12))}

if(j <= 2*s/4){sdx <- abs(rnorm(2*obs, mean=h/16, sd=h/16))}

if(j <= 1*s/4){sdx <- abs(rnorm(2*obs, mean=h/20, sd=h/20))}

}

mediax1 <- mediax[1:obs]

sdx1 <- sdx[1:obs]

mediax2 <- mediax[(obs+1):(2*obs)]

sdx2 <- sdx[(obs+1):(2*obs)]

x1 <- matrix(,nrow=n,ncol=obs)

x2 <- matrix(,nrow=n,ncol=obs)

i=1

for (i in 1:obs){

x1[,i] <- rnorm(n,mediax1[i],sdx1[i])

x1[which(x1[,i]<=0),i]<-0.001

x2[,i] <- rnorm(n,mediax2[i],sdx2[i])

x2[which(x2[,i]<=0),i]<-0.001

i=i+1

}

CVx1 <- apply(x1,2,sd)/apply(x1,2,mean)

CVx2 <- apply(x2,2,sd)/apply(x2,2,mean)

lnx1 <- log(x1)

lnx2 <- log(x2)

numHald <- ((apply(lnx1,2,mean)-apply(lnx2,2,mean)))/sqrt(((apply(lnx1,2,sd))^2+(apply(lnx2,2,sd))^2)*19/38)

Cohen <- ((apply(x1,2,mean)-apply(x2,2,mean)))/sqrt(((apply(x1,2,sd))^2+(apply(x2,2,sd))^2)*19/38)

Hedge <- Cohen*(1-3/(4*(38)-1))

Vd <- 2*n/n^2+Hedge^2/(4*n)

maxCV <- 3

for(i in 1:div){

sub_numHald <- numHald[CVx1<=maxCV-maxCV/div*(i-1) & CVx2<=maxCV-maxCV/div*(i-1)]

sub_Hedge <- Hedge[CVx1<=maxCV-maxCV/div*(i-1) & CVx2<=maxCV-maxCV/div*(i-1)]

if (length(na.omit(sub_numHald)) < 10) break

nn[i,j] <- length(na.omit(sub_numHald))

r[i,j] <- cor(sub_numHald, sub_Hedge)

CVmax[i,j] <- maxCV-maxCV/div*(i-1)

}

print(j)

j=j+1

}

LR effect size and LRI-framework

Simulation developed by Gingerich (2009). We computed *H_g_*, *LG* and *LR*. We started with a sample of 30 elements with mean= 100 and variance=1. In each generation (t), until t=200, we sampled 30 elements from a mean equal to mean in the previous time (t-1) plus a random deviation (from a normal distribution, mean = 0 and sd = 1.25), and variance=1.

set.seed(7)

N <- 200 #Number of successive samples

t.yr <- 1:N #time of successive samples (years)

t.gen <- 1 #Generation time (years)

n <- 30 #Sample size for successive samples

media <- 100 #initial value of the mean

#First, we generated a random walk of the mean of the trait.

#The trait expected variance is always 1.

#We record the information of mean and variance of the raw trait and ln-transformed varible

zbar <- zvar <- lnzbar <- lnzvar <- vector(mode="numeric", length=N)

z0 <- rnorm(n,media,1)

zbar[1] <- mean(z0)

zvar[1] <- var(z0)

lnz0 <- log(z0)

lnzbar[1] <- mean(lnz0)

lnzvar[1] <- var(lnz0)

i=2

for (i in 2:N){

media <- media+rnorm(1,0,1.25)

zi <- rnorm(n,media,1)

zbar[i] <- mean(zi)

zvar[i] <- var(zi)

lnzi <- log(zi)

lnzbar[i] <- mean(lnzi,na.rm=T)

lnzvar[i] <- var(lnzi,na.rm=T)

i=i+1

}

rm(i)

###

#Then we calculated all possible combinations of pair comparisons.

H <- vector(mode="numeric",length=(N^2+N)/2)

time <- vector(mode="numeric",length=(N^2+N)/2)

LG <- vector(mode="numeric",length=(N^2+N)/2)

Vlg <- vector(mode="numeric",length=(N^2+N)/2)

LR <- vector(mode="numeric",length=(N^2+N)/2)

Vlr <- vector(mode="numeric",length=(N^2+N)/2)

k=1

i=1

for (i in 1:N){

j=i

for(j in i:N){

lnzdiff <- lnzbar[i]-lnzbar[j] #Difference between successive means

lnzvar.w <- ((n-1)*lnzvar[i]+(n-1)*lnzvar[j])/(n+n-2) #Pooled within-sample variance

tdiff <- t.yr[j] - t.yr[i] #Time interval between samples (year)

Hn <-lnzdiff/sqrt(lnzvar.w) #Haldane numerator

H[k] <- abs(Hn/(tdiff/t.gen))) #rate in Haldanes (absolute value)

time[k] <- tdiff

zdiff <- zbar[i]-zbar[j] #Difference between successive means

zvar.w <- ((n-1)*zvar[i]+(n-1)*zvar[j])/(n+n-2) #Pooled within-sample variance

d <-zdiff/sqrt(zvar.w)*(1-3/(4*(2*n-2)-1)) #Hedge's d

Vd <- ((2*n)/(n^2)) + ((d^2)/(4*(n-2))) #Variance of Hedge's d

LG[k] <- log10(abs(d)) #LG ES

Vlg[k] <- 0.1886*Vd/(d^2) #variance of LG

h <- abs(d/(tdiff/t.gen)) #rate in h, effect size

Vh <- Vd/(tdiff^2) #variance of h

LR[k] <- log10(h) #Effect size LR

Vlr[k] <- 0.1886*Vh/(h^2) #variance of LR

k=k+1

j=j+1

}

i=i+1

}
